# Temporal and Cartographic Analyses of the Distribution within Spain of Mortality Due to Granulomatosis with Polyangiitis (1984–2016)

**DOI:** 10.3390/ijerph16081388

**Published:** 2019-04-17

**Authors:** Germán Sánchez-Díaz, Francisco Escobar, Ana Villaverde-Hueso, Manuel Posada de la Paz, Verónica Alonso-Ferreira

**Affiliations:** 1 Institute of Rare Diseases Research (IIER), Instituto de Salud Carlos III, 28029 Madrid, Spain; g.sanchez@externos.isciii.es (G.S.-D.); anavillaverde@isciii.es (A.V.-H.); mposada@isciii.es (M.P.d.l.P.); 2 Department of Geology, Geography and Environmental Sciences, University of Alcala, 28801 Alcalá de Henares, Spain; francisco.escobar@uah.es; 3 Centre for Biomedical Network Research on Rare Diseases (CIBERER), 28029 Madrid, Spain

**Keywords:** mortality, Spain, granulomatosis with polyangiitis, geographic patterns, temporal trends, latitude, mapping, Wegener

## Abstract

The aim is to conduct a descriptive, population-based study in order to assess temporal and spatial changes in mortality due to granulomatosis with polyangiitis (GPA) in Spain from 1984 to 2016. Mortality data were obtained from the Spanish Annual Death Registry. Deaths in which GPA was the underlying cause were selected using the 446.4 and M31.3 codes from the International Classification of Diseases, 9th and 10th revision. Annual average age at death and age-adjusted mortality rates were calculated. Geographic analysis was performed at municipality and district level. Variations in mortality according to the type of municipality (urban, agro-urban or rural), district and geographic location (degrees of latitude) were assessed using standardized mortality ratios (SMRs) and smoothed-SMRs. Over the whole period, 620 deaths due to GPA were identified. Age at death increased at an average annual rate of 0.78% over the period 1987–2016 (*p* < 0.05). Age-adjusted mortality rates increased by an annual average of 20.58% from 1984 to 1992, after which they fell by 1.91% a year (*p* < 0.05). The agro-urban category had the highest percentage (4.57%) of municipalities with a significantly higher GPA mortality rate than expected. Geographic analysis revealed four districts with a higher risk of death due to GPA, two in the North of Spain and two in the South. This population-based study revealed an increase in the age at death attributed to GPA. Age-adjusted mortality rates went up sharply until 1992, after which they started to decline until the end of the study period. Geographic differences in mortality risk were identified but further studies will be necessary to ascertain the reasons for the distribution of GPA disease.

## 1. Introduction

Granulomatosis with polyangiitis (GPA), formerly known as Wegener’s Granulomatosis, is a systemic necrotizing vasculitis characterized primarily by a granulomatous inflammation of small and medium vessels [1]. It typically affects the respiratory tract and kidneys leading to premature death if not treated [2]. It is considered a rare disease due to its low prevalence (less than five cases per 10,000 inhabitants) and its etiology remains unknown [3,4,5].

Mortality due to GPA is still high, especially in the first year after diagnosis, despite better treatments being available [6]. Although the death rate amongst GPA patients has been reduced since the beginning of the 21st century, it remains higher than the mortality in the general population [7]. Age at death is increasing amongst patients suffering from this rare disease due to improvement in treatments based on cyclophosphamide, glucocorticoids and rituximab [8,9].

The incidence of GPA at an international level has been increasing since the 1980s and then stabilized in the early 2000s, suggesting an increasing physician awareness following the introduction of routine anti-neutrophil cytoplasmic antibodies (ANCAs) testing [10]. Furthermore, there are considerable variations between the incidence rates in different countries [2]. In Europe, notwithstanding the fact that there are variations in methodology in the different studies, data suggest that the incidence of this disease is two to four times greater in northern than in southern countries. Previous researchers reported an annual incidence rate of 9.8 (95% CI = 7.4–12.2) per million people in Sweden, 9.3 (8.1–10.6) in Finland, 11.8 (10.7–12.9) in United Kingdom, and 7.7 (4.1–11.4) in Poland, indicating a possible spatial variation in incidence rates [11,12,13,14]. In Spain, epidemiological information about GPA is still scarce and there are no studies of mortality or other indicators at a national level. The little research so far conducted on this subject has focused on incidence rates at a provincial or local level. These studies found that Lugo province had an annual rate of 2.95 cases per million inhabitants, the city of Sabadell in Barcelona province had 4.1 cases, while the rate in Málaga province was much lower at 2.1 cases per million [15,16,17].

Geographic analyses of GPA in which populations are stratified into rural and urban areas have been conducted in several European regions and in parts of Australia [11,17,18,19]. These investigations sought to assess the differences between the incidence or prevalence of GPA in rural and urban areas. In the case of Lugo province (Spain), for example, there was higher incidence in urban areas. Several studies have also analyzed GPA by comparing incidence rates in Europe according to latitude. These found higher rates in northern than in southern European countries [20]. At a Spanish level, we thought it might be interesting therefore to observe the variation in mortality rates according to latitude, based on the assumption that northern regions will have higher rates than southern ones.

In view of the paucity of national population-based data, this study also seeks to analyze mortality due to GPA in Spain over a long period. The objectives were to: (1) evaluate the time trends in GPA mortality over a 32-year period; and (2) analyze the geographical distribution of mortality at municipal and district level, in rural vs. urban areas and according to latitude.

## 2. Materials and Methods

This research focuses on the trend over time and the geographic distribution of GPA mortality for the period 1984–2016. Mortality data were obtained from the Spanish Annual Death Registry kept by the Spanish National Statistics Institute (NSI) (Instituto Nacional de Estadística). GPA as underlying cause of each death is noted by the codes 446.4 in the International Classification of Diseases (ICD) 9th edition (period 1984 and 1998), and M31.3 in the ICD 10th edition (period 1999–2016). Underlying cause of death is defined by the World Health Organization as “the disease or injury which initiated the train of morbid events leading directly to death, or the circumstances of the accident or violence which produced the fatal injury” [21]. Population data were also obtained from the NSI and categorized by gender and age at municipal and district or *comarca* level (in Spanish the word “comarca” refers to an area or territory that contains several towns and villages but is smaller than a region).

The age-adjusted mortality rates were calculated, using the European standard population as reference, for males, females, and both sexes (expressed per 1,000,000 inhabitants). Annual trends were smoothing using the TH4253 non-parametric procedure which uses running medians to summarize overlapping segments in the time-series data leading to delete random movements [22]. Average age at death and mortality trends were evaluated using joinpoint regression models to describe changes in the time-series data where line segments are joined in the joinpoints [23].

In order to assess the variation in mortality in rural and urban areas, the Spanish municipalities were broken down into three categories. Normally, municipalities are classified as rural or urban according to population criteria [17,19]. However, this classification seems imprecise due to the varying characteristics of municipalities in Spain. Northern municipalities are usually smaller in terms of population but have more industry, while Southern municipalities tend to have larger populations and are economically more oriented towards the agricultural sector. We therefore decided to include an intermediate category for those municipalities with both urban and rural characteristics using not only population data but also the number of people working in the primary sector (PWPS), which includes activities such as agriculture, livestock, mining or fishing. The data about the sectors in which people are employed were provided by the NSI. On this basis, in this study we used the following three categories:

(a) Urban: less than 5% PWPS
(b) Agro-urban: more than 5% PWPS and more than 10,000 inhabitants
(c) Rural: more than 5% PWPS and less than 10,000 inhabitants or labor force of less than 50 people.

To assess the variation in mortality according to latitude, Spain was divided into horizontal strips of one degree of latitude. The most northerly strip was at 44° and the most southerly at 36°. The municipalities in the Canary Islands were grouped together under the same latitude strip (from 27° N to 29° N).

For disease mapping purposes, standardized mortality ratios (SMRs) were calculated by municipality using the expected GPA mortality rate in Spain for the period 1999–2016 as a reference (SMR = 1.00). Municipalities were previously divided into three categories as described above: urban, agro-urban and rural areas. Similarly, SMRs were also calculated for groups of municipalities with the same latitude in an attempt to uncover possible North-South variations in mortality.

The smoothing procedure for geographic units was carried out at *comarca* (district) level. The smoothed-SMRs for the period 1999–2016 were calculated for district using the cases observed in each district together with the expected cases, and taking the sex- and age-specific death rates for the Spanish population as a reference. We used a conditional autoregressive model based on two effects: unstructured spatial random effect and spatially structured heterogeneity [24]. As a measure of uncertainty, the Posterior Probability (PP) of each district being above average risk was computed. PP shows those districts with significantly higher (PP > 0.80) or lower (PP < 0.20) risks of death due to GPA than that expected for the country as a whole.

The statistical analyses and the smoothing procedure were performed using the Stata and R software respectively. The ArcGIS software program (Esri, Redlands, CA, USA) was used for geographic analysis and cartographic representations.

## 3. Results

**Figure 1:**
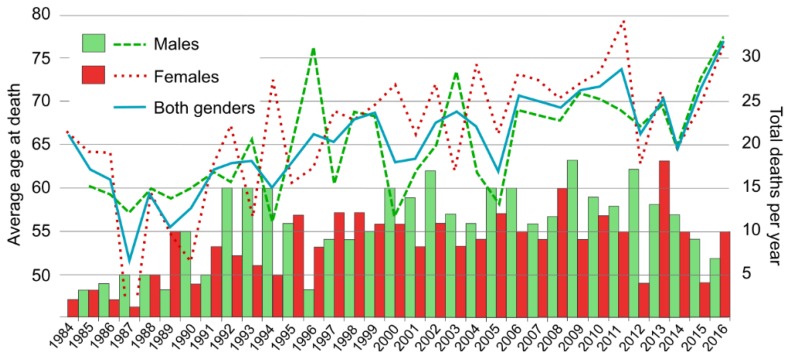
Average age at death (lines) and number of deaths (bars) due to granulomatosis with polyangiitis (GPA) in Spain by gender: 1984–2016.

Overall, we identified 620 deaths from GPA over the period 1984–2016 in Spain, of which 55.5% were males (Figure 1). The average age at death for GPA patients was 65.6 years ± 14.6 Standard Deviation (STD); 65.2 years ± 14.6 STD in males; and 65.8 years ± 14.8 STD in females. GPA age at death increased by 0.78% a year (annual percent change, APC) between 1987 and 2016 (*p* < 0.05). By sexes, age at death increased annually by 0.61% in males and 0.74% in females over the whole study period (1984–2016) (*p* < 0.05).

**Figure 2:**
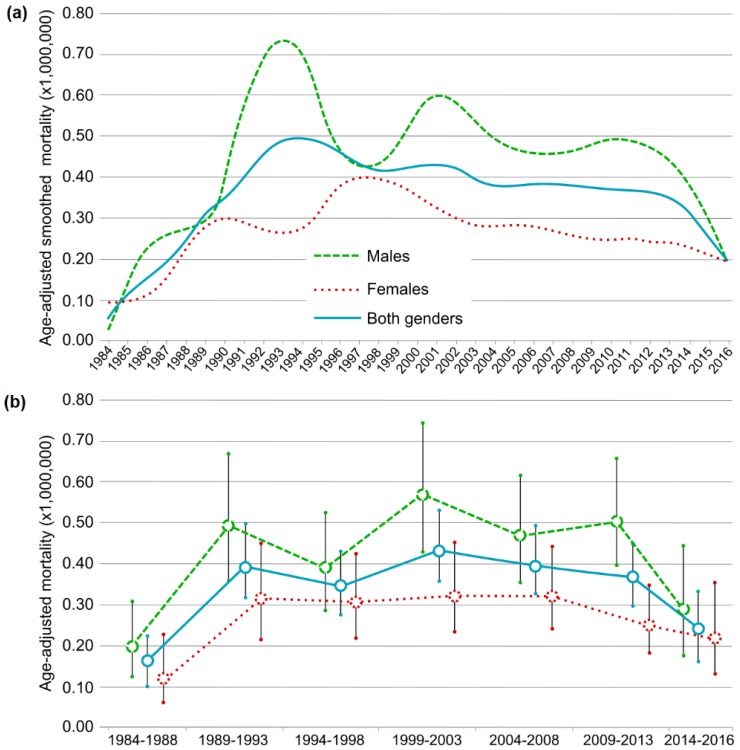
Age-adjusted mortality rates due to GPA in males, females and both genders. (**a**) Annual smoothed rates; (**b**) five-year rates (except for 2014 to 2016), bars show 95% confidence intervals.

The overall age-adjusted mortality rate for the period was 0.35 (95% Confidence Interval (CI) = 0.32–0.38) per million inhabitants: 0.44 (95% CI = 0.39–0.49) in males, and 0.28 (95% CI = 0.24–0.32) in females. Joinpoint analysis showed a 20.58% increase in the age-adjusted mortality rate between 1984 and 1992 (*p* < 0.001) and an annual fall of 1.91% from 1992 to 2016 (*p* < 0.001). Rates were higher for males than for females, and there was a significant increase of 21.57% APC (*p* < 0.001) over the period 1984–1992. From then on, the rate declined (−2.13% APC; *p* < 0.001). In females, there was a significant increase in the death rate of 36.1% APC (*p* < 0.001) from 1984 to 1989 after which it remained stable over the rest of the period. These trends for both sexes (males and females) are similar to the smoothed age-adjusted mortality rates and in the aggregation into intervals (Figure 2).

According to our classification of Spanish municipalities as urban, agro-urban or rural, 16.2% were considered as urban, 2.7% as agro-urban and 81.1% as rural (Table 1). Deaths due to GPA took place in 271 municipalities (3.34% of the total of Spanish municipalities). According to SMR by municipality, 4.57% of agro-urban municipalities had a significantly higher GPA mortality than expected for Spain as a whole, while this percentage was lower in urban and rural municipalities (Table 1). Only one urban municipality obtained a death risk lower than expected (Barcelona: SMR = 0.52; 95% CI = 0.24–0.98). However, global SMR in these categories was similar to the expected value (SMR = 1.00): SMRs due to GPA in all agro-urban areas considered as a whole were 1.25 (95% CI = 0.93–1.64), 0.87 (95% CI = 0.68–1.10) in rural and 1.00 (95% CI = 0.89–1.12) in urban.

**Figure 3:**
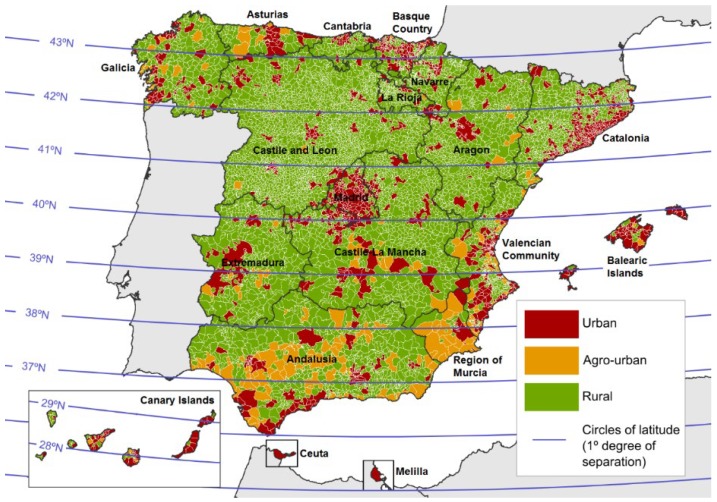
Classification of urban, agro-urban and rural municipalities in Spain. Latitude bands are shown.

Throughout the Spanish territory, while urban municipalities are frequent along the Mediterranean (Catalonia, Valencia) and Atlantic coasts (northern Spain) and in the center (around Madrid), rural municipalities tend to be concentrated in inland Spain (Figure 3). Most agro-urban municipalities are located in the South (Andalusia and Murcia). Agro-urban areas typically have a medium-sized population and a relatively strong primary sector compared with other parts of Spain.

According to SMR results for municipalities grouped within the same degree of latitude, an increased death risk due to GPA can be observed not only in the far North of the Iberian Peninsula but also in the South (degree 36° N and statistically significant: SMR = 1.79) for both sexes (Figure 3; Table 2). The lowest death rate was in the 40° N strip (SMR = 0.75 and statistically significant), which runs across the center of the Iberian Peninsula and includes the capital, Madrid. By sexes, SMR results showed a significantly higher death risk for males in the coastal areas of Andalusia in southern Spain (36° N strip): SMR = 1.91; 95% CI = 1.29–2.72), however it was not significant for women. In the southernmost latitude in Spain, the Canary Islands, there was a higher risk for both sexes together and for women separately, however these results were not significant.

**Figure 4:**
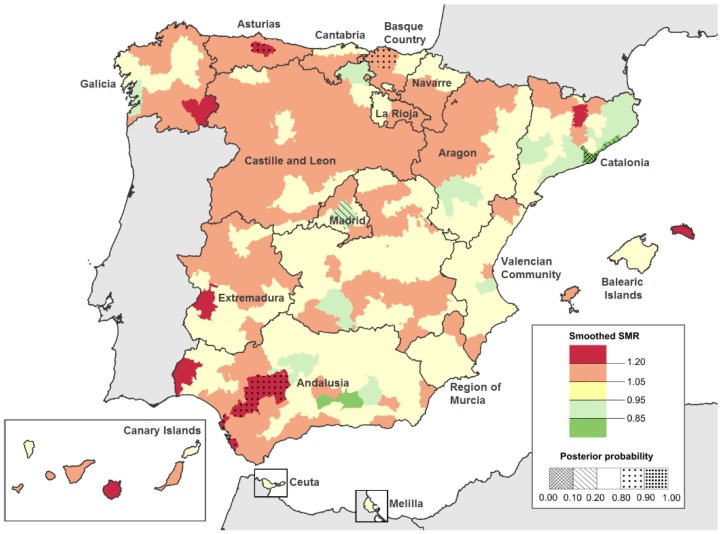
GPA Smoothed-SMR and Posterior probability (PP) by district in Spain (1999–2016, both genders). PP shows those districts with significantly higher (PP > 0.80) and lower (PP < 0.20) risk of death due to GPA than expected for Spain as a whole.

Finally, in smoothed SMRs a similar pattern can be seen as the territory gradation according to latitude: there are several areas in the North and in the South with a higher than expected death rate while in the center there were several districts with lower than expected results (Figure 4). The lowest death risks due to GPA were noted in some areas of western Galicia (Northwest) and in metropolitan areas of Madrid and Barcelona. While of the four districts in Spain with a significantly higher risk of death (PP > 0.80), two were in the North (one in Galicia and one in the Basque Country) and two were in the South (Andalusia).

## 4. Discussion

This retrospective epidemiological study on mortality due to GPA is the first of its kind in Spain at a national level and over a long period. In this paper we offer a detailed description of geographic variation in mortality rates from different perspectives so as to understand the spatial distribution of this rare disease in Spain.

The annual increase we noted in the average age at death due to GPA is in step with a similar study in England over the period 1992–2013 [25]. The increasing survival rate has been achieved thanks to quicker diagnosis, which has enabled improved treatments to alleviate damage to affected organs [9]. This has been achieved partly due to the introduction of blood tests for ANCAs, as positive levels of ANCAs proteins are related to certain forms of systemic vasculitis such as GPA disease [26,27,28,29]. Better and faster diagnosis result in increased survival of the patients affected. Other authors reported an improvement in the survival of GPA patients in recent decades associated with the use of less aggressive therapies, so reducing other complications, although this depends on the organ affected [25,30].

We found that age-adjusted mortality rates have decreased significantly since 1992. In a nationwide cohort of patients with end-stage renal disease due to GPA carried out in the United States, mortality rates decreased dramatically between 1995 and 2014, reflecting an improvement in the management of this disease over recent decades [31]. The change in the ICD code in 1999 (from the 9th to 10th edition) does not seem to have affected the trends in GPA age-adjusted mortality rates in Spain. Nevertheless, we do not know the impact of new clinical and molecular classifications for vasculitis on the ICD coding of deaths due to GPA in Spain i.e., “Revised International Chapel Hill Consensus Conference Nomenclature of Vasculitides 2012” [32]. Although our change in mortality time trend was detected in 1992, long before this nomenclature revision for GPA, we cannot rule out some effect in our data. An increase in these rates was observed in the population as a whole and in males until 1994 (the opposite was observed in women), although from then on, the rate started to fall.

Variations in mortality in urban and rural areas could help to clarify whether GPA disease is triggered by environmental factors. In a study comparing vasculitis diseases in Germany, there were higher incidence rates in cities than in rural areas [19]. In a study of the northern province of Lugo in Spain, GPA incidence was also higher in urban municipalities [17]. However, the results of similar studies in other countries have not confirmed these trends [33,34]. In our study, almost 5% of agro-urban municipalities showed significantly higher GPA mortality than expected for Spain as a whole, and this percentage was much lower in rural and urban municipalities. Agro-urban municipalities are medium-sized municipalities in terms of population (average 21,500 inhabitants) in which a substantial proportion of their citizens still work in the primary sector (more than 5%) in activities such as agriculture and livestock farming. A case-control study on this question in Norfolk (Eastern England) found a significant association between GPA and employment in farming [35]. In our study there is not sufficient ground to establish any positive association between farming in rural areas and GPA given the very low proportion of rural municipalities with higher SMR than expected. Our results are therefore not consistent with the findings obtained by Lane. Further studies must be carried out in order to find out if there is any link between mortality and farming or perhaps with the use of pesticides.

Several studies suggest that the incidence of GPA in Europe might follow a geographic pattern and decreases from North to South [36,37]. This suggests that there is a higher chance of developing GPA in northern countries perhaps due to environmental or genetic factors. Some authors highlight a possible link with the sunlight protection factor (higher in Mediterranean countries) and the benefits of Vitamin D [38]. The role of genetics in the incidence of GPA was also discussed by various authors, particularly with regard to the more frequent presence of the PTPN22 R620W polymorphism in northern European populations [39]. Another comparative study showed a higher incidence of this disease in Northern Spain than in the south of the country [15]. In our study, mortality rates did not follow any obvious latitude pattern, with higher rates in areas of both Northern and Southern Spain, so coming to the same conclusions as observed in Germany in the period 1998–1999 [36]. In addition, latitude effect could be only noticeable at an international scale, such as European, and not in a single country. It is also possible that a geographic pattern in Spain could have been weakened due to internal migratory flows over the course of history [40].

When we analyzed the results for the different Spanish districts, no clear geographical North-South pattern could be observed. Nor could we find any significant rural/urban differences. This suggests that further, in-depth analysis is required to explain the geographic distribution of GPA in Spain. Family grouping in GPA disease is infrequent except in cases of first-degree relatives, which would suggest that the disease may have environmental causes [41]. Several studies point out that higher incidences of systemic vasculitis might be associated with environmental factors, such as silica exposure [42,43]. However, everything suggests that GPA is a complex, multifactorial disease in which both genetics and the environment play a role [20,35,44].

The present study has several limitations, such as the fact that our population-based death registry does not provide personal information with regard to occupation, disease-related genetic history or personal habits. For instance, a recent Spanish study found that GPA patients were more likely to have a history of smoking than the population as a whole [45]. Findings like these make the relationship between possible triggers for the disease and likely environmental factors increasingly difficult to unravel. In addition, most GPA epidemiological studies focus on prevalence and incidence, which makes it harder to compare mortality rates with other reports not only in Spain but also in other countries. Our research consisted of a nationwide, population-based study over a long period of time of the mortality directly attributed to GPA and followed a homogeneous, standardized methodology. The increase in average age at death identified in our study is an important finding in that it allows national or regional governments to plan the health services required to improve the life of GPA patients more effectively. It is important to emphasize for example that the treatments for GPA disease can be quite costly because patients often suffer relapses [46,47].

## 5. Conclusions

In conclusion, this study has analyzed deaths due to GPA in Spain over a 32-year period. Since 1992, mortality rates have fallen and average age at death has increased due to better treatments and prompt diagnosis. We found that areas categorized as agro-urban had a greater proportion of municipalities with higher than expected mortality in comparison with urban and rural areas. We also analyzed differences in death rates according to latitude, but no spatial pattern was found. More in-depth studies are required to enable us to gain a better understanding of the etiology of GPA disease.

## Figures and Tables

**Table 1:** Distribution of municipalities categorized as urban, agro-urban or rural; number of deaths due to GPA and municipalities with higher or lower GPA mortality than expected for Spain in 1999–2016 (only statistically significant differences are shown, *p* < 0.05).

Category	Municipalities (%)	Municipalities Registering GPA Deaths	Deaths Due to GPA	Municipalities (%) with Significant SMR Due to GPA	
				Higher Than Expected	Lower Than Expected
**Urban**	1316 (16.2%)	162	300	22 (1.67%)	1 (0.08%)
**Agro-urban**	219 (2.7%)	40	52	10 (4.57%)	0
**Rural**	6588 (81.1%)	69	71	25 (0.38%)	0

**Table 2:** Number of municipalities, deaths and standardized mortality ratio (SMR) results for each degree of latitude due to GPA disease over the period 1999–2016 in Spain.

Grade of Latitude	No. Municipalities	GPA Deaths	SMR (95% CI)		
			Both Sexes	Both Sexes	Women
43° N	519	64	1.28 (0.99–1.63)	1.16 (0.79–1.63)	1.44 (0.99–2.04)
42° N	1842	50	1.05 (0.78–1.38)	1.26 (0.87–1.76)	0.77 (0.44–1.25)
41° N	2078	70	0.83 (0.65–1.05)	0.86 (0.62–1.17)	0.79 (0.53–1.13)
40° N	1507	52	0.75 (0.56–0.99)	0.73 (0.49–1.06)	0.79 (0.50–1.17)
39° N	795	40	0.90 (0.64–1.22)	0.91 (0.58–1.37)	0.88 (0.51–1.41)
38° N	579	37	0.97 (0.68–1.34)	1.00 (0.62–1.51)	0.93 (0.53–1.53)
37° N	500	41	0.88 (0.63–1.20)	0.80 (0.50–1.23)	0.99 (0.61–1.53)
36° N	215	49	1.79 (1.33–2.37)	1.91 (1.29–2.72)	1.64 (0.98–2.55)
27°–29° N	88	20	1.28 (0.78–1.98)	0.87 (0.38–1.72)	1.84 (0.95–3.22)
